# Method for Identifying Essential Proteins by Key Features of Proteins in a Novel Protein-Domain Network

**DOI:** 10.3389/fgene.2021.708162

**Published:** 2021-06-29

**Authors:** Xin He, Linai Kuang, Zhiping Chen, Yihong Tan, Lei Wang

**Affiliations:** ^1^College of Computer, Xiangtan University, Xiangtan, China; ^2^College of Computer Engineering & Applied Mathematics, Changsha University, Changsha, China

**Keywords:** essential proteins, protein-protein network, computational model, domain-domain network, protein-domain network

## Abstract

In recent years, due to low accuracy and high costs of traditional biological experiments, more and more computational models have been proposed successively to infer potential essential proteins. In this paper, a novel prediction method called KFPM is proposed, in which, a novel protein-domain heterogeneous network is established first by combining known protein-protein interactions with known associations between proteins and domains. Next, based on key topological characteristics extracted from the newly constructed protein-domain network and functional characteristics extracted from multiple biological information of proteins, a new computational method is designed to effectively integrate multiple biological features to infer potential essential proteins based on an improved PageRank algorithm. Finally, in order to evaluate the performance of KFPM, we compared it with 13 state-of-the-art prediction methods, experimental results show that, among the top 1, 5, and 10% of candidate proteins predicted by KFPM, the prediction accuracy can achieve 96.08, 83.14, and 70.59%, respectively, which significantly outperform all these 13 competitive methods. It means that KFPM may be a meaningful tool for prediction of potential essential proteins in the future.

## Introduction

Essential proteins are indispensable proteins in the reproduction and survival of organisms, and experimental results have shown that removal of essential proteins may lead to inability of organisms to survive and develop (Zhang Z. et al., 2020; Zhao et al., 2020; Meng et al., 2021). In recent years, with the rapid development of high-throughput technologies, more and more interactions between proteins have been found in *Saccharomyces cerevisiae*, and it has become a hot spot of research to identify essential proteins from large amount of known protein-protein interaction (PPI) data by adopting computational methods. Up to now, a lot of computational prediction methods have been proposed successively to infer potential essential proteins, and in general, these methods can be roughly divided into two categories. The first category of methods mainly relies on topological characteristics of PPI networks to predict essential proteins. For instance, based on the centrality-lethality rule (Jeong et al., 2001) that proteins with high degree of interconnectivity are more likely to be essential proteins than those with low degree of interconnectivity in a PPI network, a series of centrality-based methods including DC (Hahn and Kern, 2004), CC (Wuchty and Stadler, 2003), BC (Joy et al., 2005), EC (Bonacich, 1987), SC (Estrada and Rodriguez-Velazquez, 2005), and IC (Stephenson and Zelen, 1989) have been designed to identify key proteins by the interconnectivities of proteins in PPI networks, and among them, the SC method was proven to be the best (Estrada, 2010). Except for these centrality-based methods, Wang et al. (2012) presented a method named NC for detecting essential proteins based on the edge aggregation coefficients. Li et al. (2011) proposed a method called LAC to predict essential proteins by evaluating the relationship between proteins and their neighbors in the PPI network. Wang et al. (2011) put forward a model called SoECC by the correlation between PPI network proteins. Przulj et al. (2004) designed a prediction model by constructing the shortest path spanning tree for each protein in the PPI network. In the first category of methods, some topological structures of PPI networks such as the node degree of interconnectivities and common neighboring nodes have been adopted to infer key proteins, however, due to the incompleteness of PPI networks, these methods cannot achieve satisfactory prediction accuracy.

In order to overcome the limitations of the first category of methods, the second category of methods focus on predicting essential proteins by combining topological features of PPI networks and functional features of proteins extracted from the gene expression data, orthology information and the subcellular localization of proteins. For example, Lei et al. (2018c) combined topological features of PPI networks and the GO data of proteins to design a novel method called RSG for predicting essential proteins. Zhang et al. (2013) designed a method called COEWC by integrating neighborhood features of the PPI network with the gene expression data of proteins to infer key proteins. Li et al. (2012) developed a prediction model named Pec by combining the PPI network and the gene expression data of proteins. Tang et al. (2014) proposed a novel method named WDC based on the edge clustering coefficients and the Pearson correlation coefficients of proteins. Peng et al. (2012) developed a computational model called ION by integrating the protein orthology information with PPI data to predict essential proteins. Xiao et al. (2013) developed a method for predicting essential proteins by combining the PPI network with the co-expressed gene data of proteins. Ren et al. (2011) invented a method to identify key proteins by integrating PPI networks with the protein complex information. Jiang et al. (2015) integrated topological features of PPI networks with the gene expression data of proteins to design a prediction model called IEW for key protein prediction. Zhao et al. (2014) developed a computational method named POEM by combining the gene expression data of proteins with topological attributes of PPI networks. Zhong et al. (2020) developed a predictive model called JDC by combining topological characteristics of PPI networks and gene expression data of proteins. Keretsu and Sarmah (2016) used the marginal clustering coefficients and the gene expression correlation between interacting proteins to design a method for identifying protein complexes. Zz et al. (2019) proposed a method that refines PPI networks by using gene expression information and subcellular localization information. Ahmed et al. (2021) designed a predictive model called EPD-RW through incorporating PPI networks with four kinds of biological data of proteins including GO data, gene expression profiles, domain information and phylogenetic profile to infer essential proteins. Zhang et al. (2021) proposed an identification model by combining PPI networks with the gene expression profile, GO information, subcellular localization information, and orthology data of proteins to detect essential proteins. Lei et al. (2018a) combined the gene expression data, subcellular location and protein complex information of proteins with the topological characteristics of PPI networks to develop a key protein identification algorithm FPE. Zhao et al. (2019) designed an iterative method called RWHN by integrating the PPI network with domains, subcellular location and homology information of proteins to identify essential proteins. Lei et al. (2018b) designed a novel calculation model named AFSO_EP to identify essential proteins by combining PPI networks with the gene expression, GO annotation and subcellular location information of proteins. Zhang et al. (2019) proposed a predictive model called TEGS by combining multiple functional features including the subcellular location data and gene expression data of proteins with topological features of PPI networks. Li et al. (2020) put forward a prediction model named CVIM by combing gene expressions data and orthologous information of proteins with PPI networks to infer essential proteins.

Experimental results have demonstrated that the second category of methods can achieve better prediction performance than the first category of methods by integrating biological characteristics of proteins and topological characteristics of PPI networks, and it is useful to adopt the biological characteristics of proteins to compensate for the incompleteness of the PPI data. Hence, in order to further improve the accuracy of prediction models, in this paper, we extracted some new topological features from a newly constructed protein-domain network and some new functional features of proteins from the domain data, gene expression data, and orthologous information of proteins etc., based on which, a novel identification model called KFPM was proposed to infer potential essential proteins. Different from existing models, in KFPM, the gene expression data of protein will be processed first by adopting the Pearson Correlation Coefficient (PCC) (Horyu and Hayashi, 2013), and then, an improved Criteria Importance Though Intercrieria Correlation algorithm (CRITIC) (Zhang B. et al., 2020) will be applied to effectively combine multiple biological features of proteins by the contrast strength of features and the conflicts between features, based on which, a novel distribution rate network is constructed and an improved PageRank algorithm will be designed to identify potential essential proteins. Finally, we compared KFPM with 13 advanced methods including DC (Hahn and Kern, 2004), CC (Wuchty and Stadler, 2003), BC (Joy et al., 2005), EC (Bonacich, 1987), SC (Estrada and Rodriguez-Velazquez, 2005), IC (Stephenson and Zelen, 1989), NC (Wang et al., 2012), CoEWC (Zhang et al., 2013), Pec (Li et al., 2012), ION (Peng et al., 2012), POEM (Zhao et al., 2014), TEGS (Zhang et al., 2019), and CVIM (Li et al., 2020). And experimental results showed that KFPM outperformed all these competitive state-of-the-art predictive methods as a whole.

## Materials and Methods

### Experimental Data

In this section, In order to evaluate the prediction accuracy of KFPM, known protein-protein interactions (PPI) would be downloaded first from the saccharomyces cerevisiae related public databases including DIP database (Xenarios et al., 2002), the Krogan database (Krogan et al., 2006), and the Gavin database (Gavin et al., 2006), respectively. As illustrated in Table 1, after filtering out repetitive interactions, we finally obtained 5,093 proteins and 24,743 interactions from the DIP database, 3,672 proteins and 14,317 interactions from the Krogan database, and 1,855 proteins and 7,669 interactions from the Gavin database. Next, we downloaded 1,107 domains from the Pfam (Bateman et al., 2004) database as well. Therefore, we constructed a (5,093+1,107) × (5,093+1,107), a (3,672+1,107) × (3,672+1,107) and a (1,855+1,107) × (1,855+1,107) dimensional networks by combining the datasets downloaded from the DIP, the Krogan and the Gavin databases with the dataset downloaded from the Pfam database separately. Moreover, we downloaded the gene expression data for calculating the initial protein scores from the Tu-BP database (Tu et al., 2005). Gene expression data contains 6,776 lines with length of 36, and each line represents the corresponding expression data of a different gene. Through comparison, we found that in datasets downloaded from the DIP and the Gavin databases, the number of proteins containing the gene expression data is more than 95%. Additionally, we downloaded orthologous information of proteins from the InParanoid database (Gabriel et al., 2010) and subcellular localization data of proteins from the COMPART-MENTS databases (Binder et al., 2014) to calculate initial scores for proteins, and as a result, we derived eleven subcellular locations such as the Mitochondrion, Peroxisome, Plasma, Extracellular, Endosome, Vacuole, Endoplasmic, Cytosol, Golgi, and Cytoskeleton Nucleus, that are related to essential proteins. Finally, a benchmark dataset for testing different prediction models was downloaded from the following four databases such as MIPS (Mewes et al., 2006), SGD (Cherry et al., 1998), DEG (Zhang and Lin, 2009), and SGDP (Saccharomyces Genome Deletion Project, 2012), which contains 1,293 key proteins. In this paper, we would provide comparison results based on datasets downloaded from the DIP and the Krogan databases in detail, and introduce briefly the experimental results based on the dataset downloaded from the Gavin database instead.

**TABLE 1:** The information of the DIP, Krogan and Gavin database.

Database	Proteins	Interactions	Essential proteins
DIP	5,093	24,743	1,167
Krogan	3,672	14,317	929
Gavin	1,855	7,669	714

**FIGURE 1:**
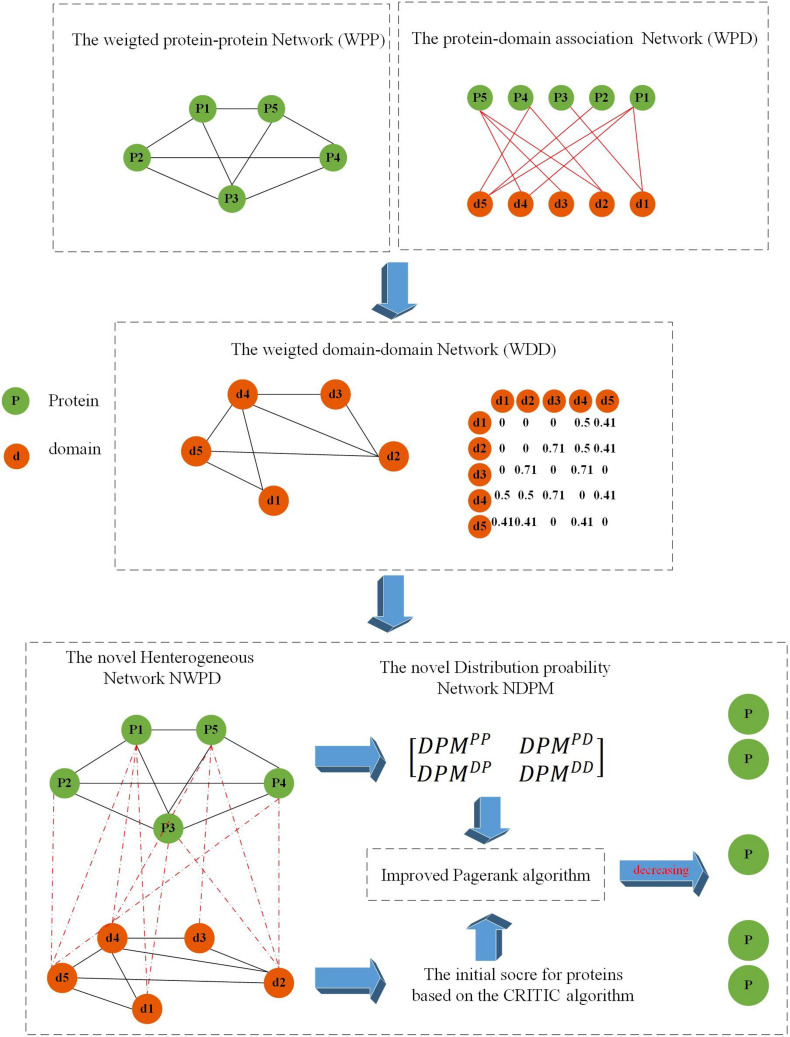
Flowchart of KFPM.

As shown in Figure 1, the flowchart of KFPM consists of the following four major steps:

Step 1: Based on known PPI dataset downloaded from a given public database, an original PPI network will be constructed first. And then, based on key topological characteristics of the original PPI network, weights between protein nodes will be calculated and adopted to transform the original PPI network to a weighted PPI network.
Step 2: Next, based on known relationships between proteins and domains, a weighted domain-domain network and an original protein-domain network will be constructed sequentially. And then, a novel heterogeneous protein-domain network will be established by integrating these three newly constructed networks such as the weighted PPI network, the weighted domain-domain network and the original protein-domain network.
Step 3: Moreover, an improved CRITIC algorithm will be applied to effectively integrate multiple biological features of proteins with key topological features extracted from the heterogeneous protein-domain network to calculate initial scores for proteins and domains.
Step 4: Finally, a novel transition probability matrix will be obtained, and then, through combining initial scores of proteins and domains with the transition probability matrix, a new iterative algorithm will be designed to identify potential essential proteins based on the PageRank algorithm.

### Construction of the Weighted PPI Network

For convenience, let *P* = {*p*_1_,*p*_2_,⋯,*p*_*N*_,} denote the set of different proteins downloaded from a given public database, and for a pair of proteins *p_u_* and *p_v_* in *P*, if there is a known interaction between them, we define that there is an edge *e*(*p*_*u*_,*p*_*v*_) = 1. Hence, let *E* represent the set of edges between proteins in *P*, Then it is obvious that we can obtain an original PPI network *PPIN* = (*P*,*E*).

Additionally, inspired by the assumption that degrees of connections between essential proteins are mostly higher than degrees of connections between non-essential proteins (Zhang et al., 2016), for any two given protein nodes *p_u_* and *p_v_* in *PPIN*, it is obvious that we can estimate the degree of relationship between them according to the following equation (1):

(1) $$WPP\left(p_{u},p_{v}\right)=\;\;\left\{ \begin{array}{l} \frac{{\left|NG\left(p_{u}\right)\cap NG\left(p_{v}\right)\right|}^{2}}{\left(\left|NG\left(p_{u}\right)\right|+1\right)\times \left(\left|NG\left(p_{v}\right)\right|+1\right)}\;if\;e(p_{u},p_{v})=1\\ 0\;\;\;\;\;\;\;\;\;otherwise \end{array}\right.$$

Here, *NG*(*p*_*u*_) represents the set of neighboring nodes of *p_u_* in *PPIN*, |*NG*(*p*_*u*_)| denotes the total number of neighboring nodes of *p_u_* in *PPIN*, and *NG*(*p*_*u*_)∩*NG*(*p*_*v*_) means the set of common neighboring nodes of both *p_u_* and *p_v_* in *PPIN*. Obviously, according to above Eq. 1, it is easy to obtain a *N*×*N* dimensional adjacency matrix *WPP*, based on which, we can obtain a weighted PPI network easily as well.

### Construction of the Heterogeneous Protein-Domain Network

In this section, we will download the domain set of proteins *D* = {*d*_1_,*d*_2_,⋯,*d*_*M*_} from the Pfam database (Bateman et al., 2004), based on which, an initial protein-domain interaction network will be constructed as follows: for any given *p*_*u*_ ∈ *P* and domain *d_v_* ∈ *D*, if there is *p*_*u*_ ∈ *d_v_*, we define that there is an edge existing between them. Thereafter, it is easy to see that we can obtain an initial protein-domain interaction network and a *N*×*M* dimensional adjacency matrix *WPD* as follows: for any given *p*_*u*_ ∈ *P* and domain *d_v_* ∈ *D*, if there is an edge between them, then there is *WPD*(*p*_*u*_,*d*_*v*_) = 1, otherwise there is *WPD*(*p*_*u*_,*d*_*v*_) = 0.

Moreover, for any two given domains *d_u_* and *d_v_*, let *N*(*d*_*u*_) and *N*(*d*_*v*_) represent the number of proteins belonging to *d_u_* and *d_v_* separately, *N*(*d*_*u*_)∩*N*(*d*_*v*_) denote the number of proteins belonging to both*d_u_* and *d_v_* simultaneously, then we can calculate the weight between *d_u_* and *d_v_* according to the following Eq. 2:

(2) W⁢D⁢D⁢(du,dv)={N⁢(du)∩N⁢(dv)|N⁢(du)|×|N⁢(dv)|⁢i⁢f⁢|N⁢(du)|>0⁢a⁢n⁢d⁢|N⁢(dv)|>0 0              o⁢t⁢h⁢e⁢r⁢w⁢i⁢s⁢e

Based on above Eq. 2, it is obvious that we can further obtain a *M*×*M* dimensional adjacency matrix *WDD*. And then, through combining above obtained *N*×*N* dimensional adjacency matrix *WPP*, *N*×*M* dimensional adjacency matrix *WPD* and *M*×*M* dimensional adjacency matrix *WDD*, wen can obtain a new (*M* + *N*)×(*M* + *N*) dimensional adjacency matrix *NWPD* as follows:

(3) $$N\,W\,P\,D=\left[\begin{array}{cc} W\,P\,P & W\,P\,D\\ W\,P\,D^{T} & W\,D\,D \end{array}\right]$$

### Calculation of Initial Scores for Proteins and Domains

In order to reduce the negative impact of false positives, in this section, we will adopt topological and functional characteristics of proteins to calculate initial scores for proteins. For any given protein*p_u_*, let *I*(*p*_*u*_) denote the orthologous information of *p_u_*, then we can obtain the orthologous score *BIO*_*I*(*p*_*u*_) of *p_u_* as follows:

(4) $$B\,I\,O\,\_\,I\,\left(p_{u}\right)=\frac{I\,\left(p_{u}\right)}{m\,a\,x_{p_{v}\in P}\,\left(I\,\left(p_{v}\right)\right)}$$

Moreover, considering that gene expression refers to the process of synthesizing protein under the guidance of genes, and Pearson correlation coefficient (PCC) is suitable for measuring the degree of linear correlation between two vectors, hence, for any two given proteins *p_u_* and *p_v_*, it is obvious that we can implement PCC on gene expressions of these two proteins to calculate the similarity between them as follows:

(5) $$P\,C\,C\,\left(p_{u},p_{v}\right)=\frac{1}{n-1}\,\sum_{i=1}^{n}\left(\frac{E\,x\,p\,\left(p_{u},i\right)-\bar{E\,x\,p\,\left(p_{u}\right)}}{\sigma \,\left(p_{u}\right)}\right)\,\left(\frac{E\,x\,p\,\left(p_{v},i\right)-\bar{E\,x\,p\,\left(p_{v}\right)}}{\sigma \,\left(p_{v}\right)}\right)$$

Here, *Exp*(*p*_*u*_,*i*)represents the expression level of *p_u_* at the *i*^*th*^ time node. $\bar{E\,x\,p\,\left(p_{u}\right)}$ denotes the average gene expression value of *p_u_*, and σ(*p*_*u*_) is the standard deviation of gene expressions of *p_u_*. Therefore, we can obtain a gene expression based functional characteristic of *p_u_* as follows:

(6) $$B\,I\,O\,\_\,E\,x\,p\,\left(p_{u}\right)=\sum_{p_{v}\,\epsilon \,N\,G\,\left(p_{u}\right)}P\,C\,C\,\left(p_{u},p_{v}\right)$$

Next, based on subcellular localizations of proteins, for any given protein *p_u_*, let *Sub*_(*p*_*u*_)_ represent the set of subcellular localizations associated with *p_u_*, we can as well obtain a subcellular localization based functional characteristic of *p_u_* as follows:

(7) $$B\,I\,O\,\_\,s\,u\,b\,\left(p_{u}\right)=\sum_{i\,\epsilon \,S\,u\,b_{\left(p_{u}\right)}}E\,v\,e_{s\,u\,b}\,\left(i\right)$$

Where,

(8) $$E\,v\,e_{s\,u\,b}\,\left(i\right)=\frac{N_{s\,u\,b}\,\left(i\right)}{A\,v\,e_{s\,u\,b}}$$

(9) $$A\,v\,e_{s\,u\,b}=\frac{\sum_{i=1}^{N_{s\,u\,b}}N_{s\,u\,b}\,\left(i\right)}{N_{s\,u\,b}}$$

Here, *N*_*sub*_ means the number of all subcellular localizations of proteins and *N_sub_*(*i*) represents the number of proteins associated with the *i*^*th*^ subcellular localization.

In KFPM, We apply an improved CRITIC method, which can be used to measure weights of different characteristics based on the contrast strengths of characteristics and the conflicts between characteristics, to integrate three kinds of biological characteristics obtained above to calculate final biological feature scores for proteins as follows:

First, let *C_j_* denote the amount of information contained in the *j*^*th*^ biological feature of protein, where *C_j_* can be expressed as follows:

(10) $$C_{j}=\sigma_{j}\,\sum_{i=1}^{n}\left(1-\left|r_{i\,j}\right|\right)$$

Here, *r*_*ij*_ represents the correlation coefficient between biological characteristics *i* and *j*. σ_*j*_ represents the standard deviation of the *j*^*th*^ biological feature. Obviously, the greater the value of *C_j_*, the greater the amount of information contained in the *j*^*th*^ biological feature. Therefore, the objective weight *w_j_* of the *j*^*th*^ biological feature can be defined as follows:

(11) $$W_{j}=\frac{C_{j}}{\sum_{j=1}^{n}C_{j}}$$

Hence, based on three kinds of biological characteristics obtained above, the final biological feature score of protein *p*_*u*_can be calculated as follows:

(12) $$B\,I\,O\,\left(p_{u}\right)=\sum_{j=1}^{n}w_{j}\,B\,F\,\left(p_{u}\right)$$

(13) $$\text{Where}\,B\,F\,\left(p_{u}\right)=\left(B\,I\,O\,\_\,I\,\left(p_{u}\right),B\,I\,O\,\_\,E\,x\,p\,\left(p_{u}\right),B\,I\,O\,\_\,s\,u\,b\,\left(p_{u}\right)\right)$$

Based on above formula (12), we have obtained biological feature scores for proteins, next, for any given protein*p_u_*, we will further calculate its topological feature score based on the topological structure of the newly constructed heterogeneous protein-domain network as follows :

(14) $$T\,O\,P\,\left(p_{u}\right)=\frac{\sum_{v\,\epsilon \,N\,G\,\left(p_{u}\right)}\left|N\,G\,\left(p_{u}\right)\cap N\,G\,\left(p_{v}\right)\right|}{\left|N\,G\,\left(p_{u}\right)\right|}$$

Where |*NG*(*p*_*u*_)∩*NG*(*p*_*v*_)| denotes the number of elements in the set of *NG*(*p*_*u*_)∩*NG*(*p*_*v*_) and |*NG*(*p*_*u*_)|denotes the number of nodes in *NG*(*p*_*u*_).

Therefore, through combining the topological feature and biological feature of *p_u_*, we can define an unique final score for *p_u_* as follows:

(15) $$S_{0}\,\left(p_{u}\right)=B\,I\,O\,\left(p_{u}\right)\times \theta +\left(1-\theta \right)\times T\,O\,P\,\left(p_{u}\right)$$

Here, θ ∈ (0,1) is a parameter of weight factor.

Additionally, in a similar way, for any given domain *d_u_*, we can as well calculate an initial topological feature score for it as follows:

(16) $$S\,\left(d_{u}\right)=\sum_{p\in d_{u}}S_{0}\,\left(p\right)$$

Since the numbers of proteins in different domains are quite different, which lead to big difference between scores of domains obtained by above formula (16), therefore, after normalization, we can obtain the final topological feature score of *d_u_* as follows:

(17) $$S_{0}\,\left(d_{u}\right)=\frac{S\,\left(d_{u}\right)}{m\,a\,x_{1\leq j\leq N}\,S\,\left(d_{j}\right)}$$

### Design of KFPM

First, for any two given proteins *p_u_* and *p_v_* in the heterogeneous protein-domain network, let $P\,P\,N\,\left(p_{u},p_{v}\right)=\frac{W\,P\,P\,\left(p_{u},p_{v}\right)}{{\left(1+m\,a\,x\,\left(W\,P\,P\,\left(p_{u},p_{v}\right)\right)\right)}^{2}}$, it is obvious that *w*e can obtain a distribution probability of *p_u_* to *p_v_* as follows:

(18) $$D\,P\,M^{P\,P}\,\left(p_{u},p_{v}\right)=\left\{ \begin{array}{c} \frac{P\,P\,N\,\left(p_{u},p_{v}\right)}{\sum_{j}P\,P\,N\,\left(p_{u},p_{j}\right)}\times S_{0}\,\left(p_{v}\right),i\,f\,P\,P\,N\,\left(p_{u},p_{v}\right)\neq 0\\ 0\;o\,t\,h\,e\,r\,w\,i\,s\,e \end{array}\right.$$

Next, for any given protein *p_u_* and domain *d_v_*, let $P\,D\,N\,\left(p_{u},d_{v}\right)=\frac{W\,P\,D\,\left(p_{u},d_{v}\right)}{{\left(1+\text{max}\,\left(W\,P\,D\,\left(p_{u},d_{v}\right)\right)\right)}^{2}}$, it is obvious that *w*e can obtain a distribution probability of *p_u_* to *d_v_* as follows:

(19) $$D\,P\,M^{P\,D}\,\left(p_{u},d_{v}\right)=\left\{ \begin{array}{c} \frac{P\,D\,N\,\left(p_{u},d_{v}\right)}{\sum_{j}P\,D\,N\,\left(p_{u},d_{j}\right)}\times S_{0}\,\left(d_{v}\right),i\,f\,P\,D\,N\,\left(p_{u},d_{v}\right)\neq 0\\ 0\;o\,t\,h\,e\,r\,w\,i\,s\,e \end{array}\right.$$

Similarly, for any given domain *d_u_* and protein *p_v_*, *w*e can obtain a distribution probability of *d_u_* to *p_v_* as follows:

(20) $$D\,P\,M^{D\,P}\,\left(d_{u},p_{v}\right)=\left\{ \begin{array}{c} \frac{P\,D\,N^{T}\,\left(d_{u},p_{v}\right)}{\sum_{j}P\,D\,N^{T}\,\left(d_{u},p_{j}\right)}\times S_{0}\,\left(p_{v}\right),i\,f\,P\,D\,N^{T}\,\left(d_{u},p_{v}\right)\neq 0\\ 0\;o\,t\,h\,e\,r\,w\,i\,s\,e \end{array}\right.$$

For any given domain *d_u_* and domain *d_v_*, let $D\,D\,N\,\left(d_{u},d_{v}\right)=\frac{W\,D\,D\,\left(d_{u},d_{v}\right)}{{\left(1+\text{max}\,\left(W\,D\,D\,\left(d_{u},d_{v}\right)\right)\right)}^{2}}$, *w*e can obtain a distribution probability of *d_u_* to *d_v_* as follows:

(21) $$D\,P\,M^{D\,D}\,\left(d_{u},d_{v}\right)=\left\{ \begin{array}{c} \frac{P\,D\,D\,\left(d_{u},d_{v}\right)}{\sum_{j}P\,D\,D\,\left(d_{u},d_{j}\right)}\times S_{0}\,\left(d_{v}\right),i\,f\,D\,D\,N\,\left(d_{u},d_{v}\right)\neq 0\\ 0\;o\,t\,h\,e\,r\,w\,i\,s\,e \end{array}\right.$$

Hence, based on above description, we can obtain a novel distribution probability matrix NDPM as follows:

(22) $$N\,D\,P\,M=\left[\begin{array}{cc} D\,P\,M^{P\,P} & D\,P\,M^{P\,D}\\ D\,P\,M^{D\,P} & D\,P\,M^{D\,D} \end{array}\right]$$

Based on above formula (22), let *S*_(*t*)_denote critical scores of proteins obtained at the *t*^*th*^ round of iteration, then we can calculate the final critical scores of proteins by an improved PageRank algorithm according to the following Eq. 23:

(23) $$S_{\left(t+1\right)}=\alpha \times N\,D\,P\,M\times S_{\left(t\right)}\,\left(1-\alpha \right)\times S_{0}$$

Here,α ∈ (0,1) is a parameter used to adjust the iterative ratio.

Based on the above descriptions, the process of KFPM can be described in detail as follows:

**Algorithm:** KFPM

Input: Original PPI network, orthologous data, subcellular data, gene expression data and domain data, iteration termination condition ε, parameter α and θ.
Output: Final critical scores of proteins.
Step 1: Establishing the heterogeneous protein-domain network according to formulas (1)–(3);
Step 2: Calculating initial scores of proteins and domains in the heterogeneous protein-domain network according to formulas (4)–(17);
Step 3: Establishing the transition probability matrix *NDPM* according to formulas (18)–(22);
Step 4: Computing *S_(t+1)_* by equation (23), let *t* = *t*+1;
Step 5: Repeating step4 until ||*S*_(*t + 1*)_−*S*_(*t*)_||^2^ < ε;
Step 6: Outputting the top *k*% predicted proteins in the descending order.

## Results

### Comparison Between KFPM and Representative Methods

**FIGURE 2:**
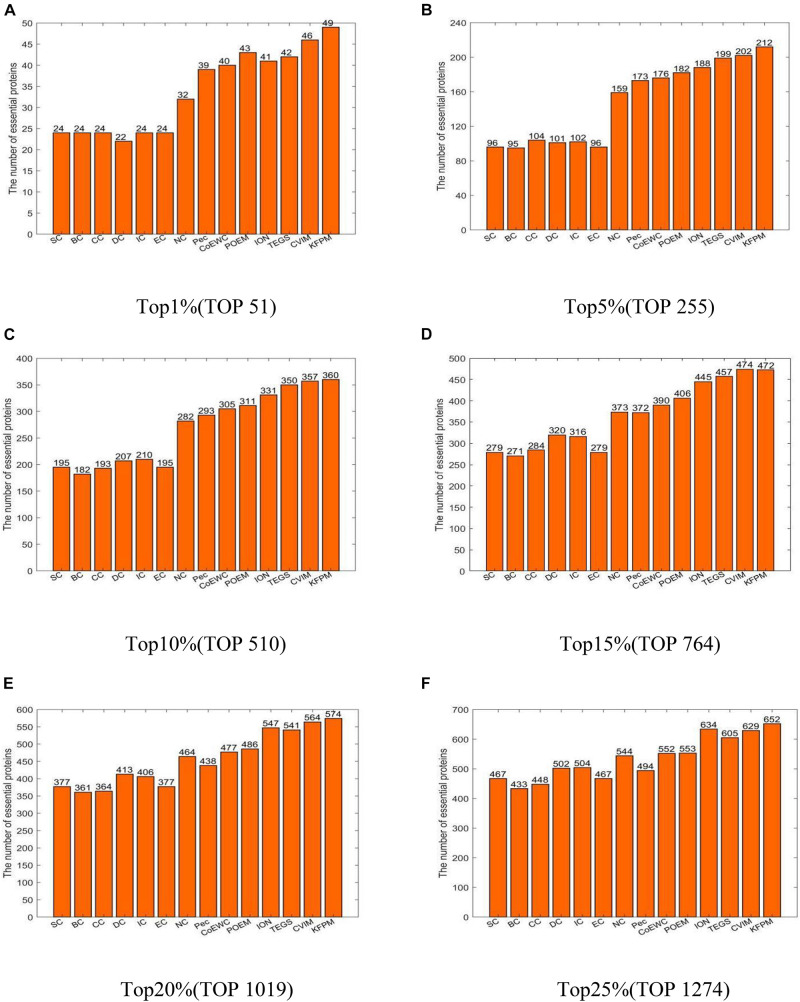
Performance comparison between KFPM and 13 competitive methods based on the DIP database **(A)** Top 1% ranked proteins. **(B)** Top 5% ranked proteins. **(C)** Top 10% ranked proteins. **(D)** Top 15% ranked proteins. **(E)** Top 20% ranked proteins. **(F)** Top 25% ranked proteins.

**FIGURE 3:**
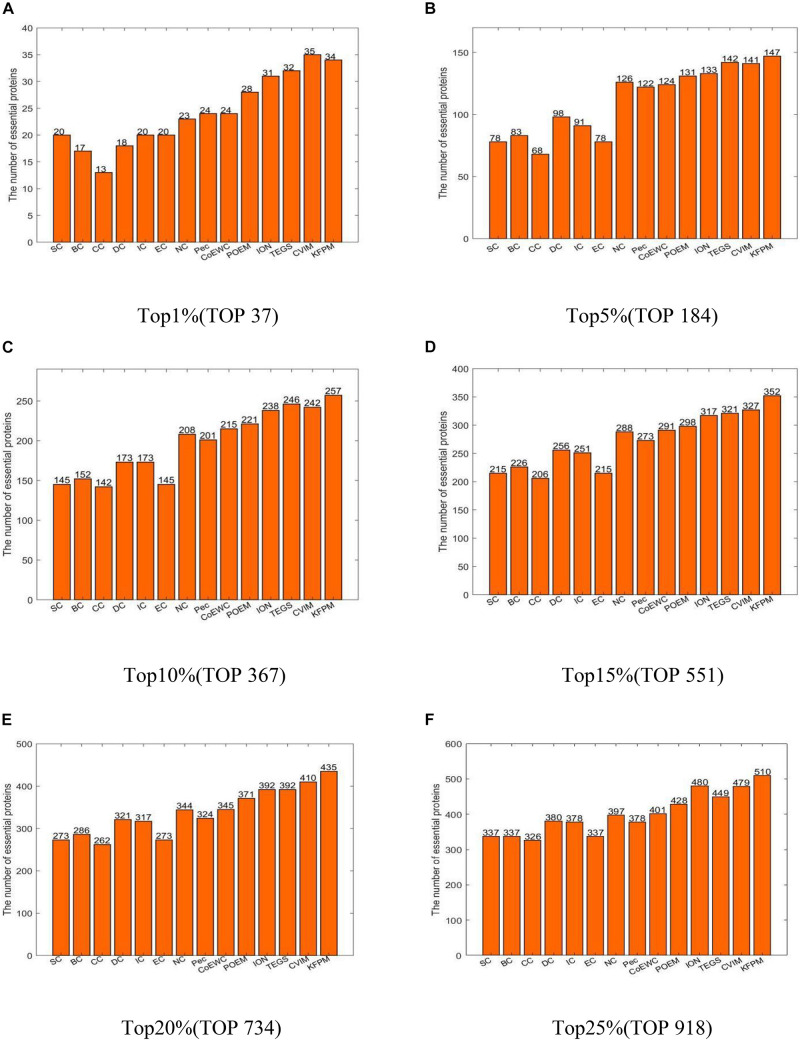
Performance comparison between KFPM and 13 competitive methods based on the Krogan database **(A)** Top 1% ranked proteins. **(B)** Top 5% ranked proteins. **(C)** Top 10% ranked proteins. **(D)** Top 15% ranked proteins. **(E)** Top 20% ranked proteins. **(F)** Top 25% ranked proteins.

In this section, we will compare KFPM with 13 state-of-the-art predictive methods based on the DIP and Krogan databases separately. Figure 2 illustrates experimental results based on the DIP database, from which, it can be seen that KFPM can achieve predictive accuracy of 96.08, 83.14, 70.59, 61.78, 56.33, and 51.18% in top 1, 5, 10, 15, 20, and 25% predicted proteins, respectively, which are better than all 13 competitive methods, except in the top 15% predicted proteins, is a little lower than CVIM. Figure 3 shows experimental results based on the Krogan database, from which, it can be seen that KFPM can achieve predictive accuracy of 91.89, 79.89, 70.03, 63.88, 59.26, and 55.56% in top 1, 5, 10, 15, 20, and 25% predicted proteins separately, which are better than all 13 competitive methods as well, except in the top 1% predicted proteins, is a little lower than CVIM. Hence, from above two kinds of experimental results, as a whole, we can conclude that the prediction performance of KRPM is better than all these 13 state-of-the-art methods.

### Validation With Jackknife Methodology

**FIGURE 4:**
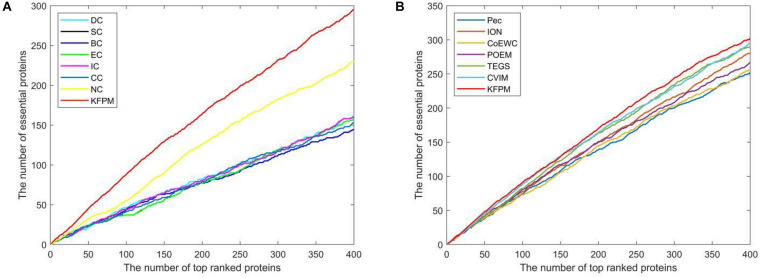
Performance comparison between KFPM and 13 state-of-the-art methods based on the method of Jackknife under the DIP database. **(A)** Comparison between KFPM and DC, SC, BC, EC, IC, CC, NC. **(B)** Comparison between KFPM and Pec, CoEWC, POEM, ION, TEGS, CVIM.

**FIGURE 5:**
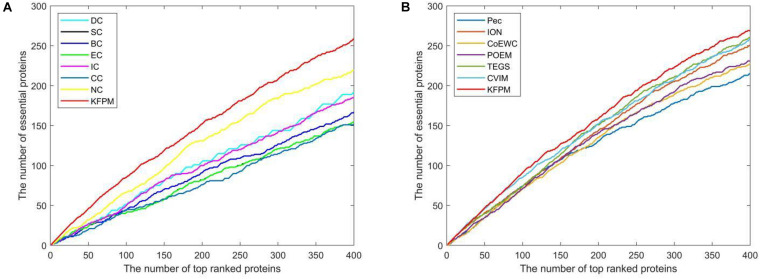
Performance comparison between KFPM and 13 state-of-the-art methods based on the method of Jackknife under the Krogan database. **(A)** Comparison between KFPM and DC, SC, BC, EC, IC, CC, NC. **(B)** Comparison between KFPM and Pec, CoEWC, POEM, ION, TEGS, CVIM.

The method of Jackknife (Holman et al., 2009) can effectively estimate the advantages and disadvantages of essential protein prediction models. Therefore, in this section, we will further utilize the method of Jackknife to compare KFPM with 13 competitive methods. Figure 4 shows the comparison result based on top 400 predicted proteins under the DIP dataset. From observing Figures 4A,B, it is easy to see that the prediction performance of KFPM is not only better than the first category of methods that are based on topological features of PPI networks only, such as DC, SC, BC, EC, IC, CC, and NC, but also better than the second category of methods that are based on the combination of biological data of proteins and PPI networks, such as Pec, CoEWC, POEM, ION, TEGS, and CVIM, simultaneously. Especially, comparing with CVIM that can achieve the best predictive performance in all these competitive methods, although the performance curves of KFPM and CVIM overlap at some times, but with the number of candidate proteins increasing, the prediction performance of KFPM will become higher and higher than CVIM. Figure 5 illustrates the comparison result based on top 600 predicted proteins under the Krogan dataset. From observing Figures 5A,B, it is obvious that KFPM can achieve better performance than both the first category of methods such as DC, SC, BC, EC, IC, CC, and NC, and the second category of methods such as Pec, CoEWC, POEM, ION, TEGS, and CVIM, as well. Hence, based on above description, we can conclude that the detective ability of KFPM is superior to all these 13 existing advanced methods.

### Difference Analysis of KFPM and Competitive Methods

**FIGURE 6:**
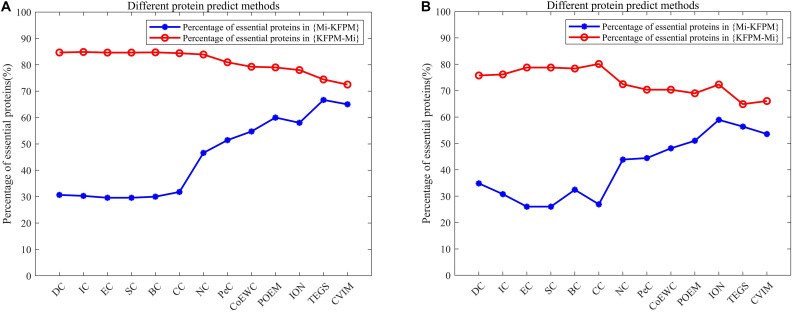
Intuitive comparison of predictive performance between KFPM and 13 competitive methods. The *X*-axis represents 13 methods. The *Y*-axis denotes the percentage of essential proteins in {KFPM−Mi} or {Mi−KFPM}.

In order to better analyze the difference and uniqueness of KFPM and state-of-the-art predictive methods, in this section, we will compare KFPM with 13 competitive methods based on top 200 predicted proteins under the DIP and Krogan databases, respectively. Comparison results are shown in Tables 2, 3, where Mi represents one of these 13 predictive methods, |KFPM∩Mi| represents the number of common essential proteins recognized by both KFPM and Mi. |KFPM−Mi| denotes the number of essential proteins that were detected by KFPM but not by Mi. {KFPM−Mi} is the set of essential proteins predicted by KFPM but ignored by Mi. {Mi−KFPM} is the set of essential proteins predict by Mi but ignored by KFPM. From observing Tables 2, 3, we can see that the proportion of key proteins in {KFPM−Mi} is higher than the percentage of key proteins in {Mi−KFPM}, which means that KFPM can screen out more essential proteins that are not found by competing methods. Figure 6 shows the superiority of KFPM more intuitively.

**TABLE 2:** Commonalities and differences between KFPM and 13 competitive methods based on top 200 ranked proteins under the DIP database.

Different methods (Mi)	| KFPM∩Mi|	| KFPM-Mi|	Percentage of key proteins in {KFPM-Mi}	Percentage of key proteins in {Mi-KFPM}
DC	37	163	84.66%	30.67%
IC	35	165	84.85%	30.30%
EC	31	169	84.62%	29.59%
SC	31	169	84.62%	29.59%
BC	30	170	84.71%	30.00%
CC	27	173	84.39%	31.79%
NC	82	118	83.90%	46.61%
Pec	95	105	80.95%	51.43%
CoEWC	94	106	79.25%	54.72%
POEM	100	100	79.00%	60.00%
ION	100	100	78.00%	58.00%
TEGS	110	90	74.44%	66.67%
CVIM	120	80	72.50%	65.00%

**TABLE 3:** Commonalities and differences between KFPM and 13 competitive methods based on top 200 ranked proteins under the Krogan database.

Different methods (Mi)	| KFPM∩Mi|	| KFPM-Mi|	Percentage of key proteins in {KFPM-Mi}	Percentage of key proteins in {Mi-KFPM}
DC	68	132	75.76%	34.85%
IC	70	130	76.15%	30.77%
EC	54	146	78.77%	26.03%
SC	54	146	78.77%	26.03%
BC	52	148	78.38%	32.43%
CC	44	156	80.13%	26.92%
NC	102	98	72.45%	43.88%
Pec	92	108	70.37%	44.44%
CoEWC	92	108	70.37%	48.15%
POEM	100	100	69.00%	51.00%
ION	88	112	72.32%	58.93%
TEGS	106	94	64.89%	56.38%
CVIM	144	56	66.07%	53.57%

### Validation by Receiver Operating Characteristic Curve

**FIGURE 7:**
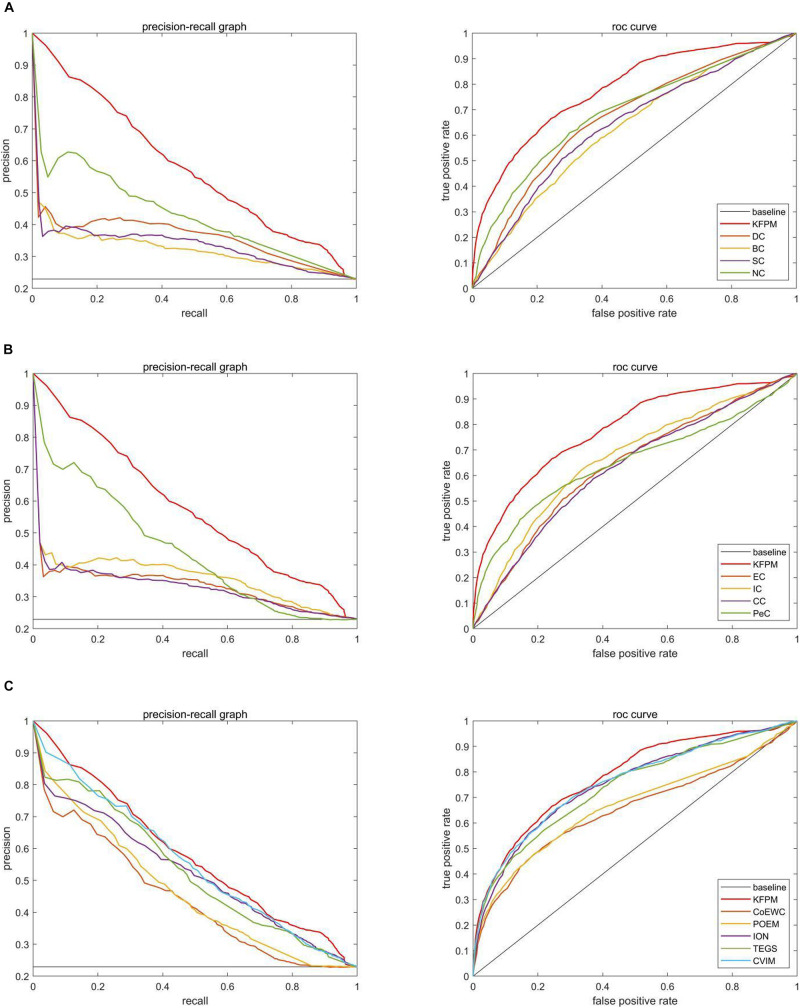
Comparison of PR curves and ROC curves between KFPM and 13 competing methods based on the DIP database. **(A)** PR curves and the ROC curves of DC, BC, SC, and NC. **(B)** PR curves and ROC curves of EC, IC, CC and Pec. **(C)** PR curves and ROC curves of CoEWC, POEM, ION, TEGS, and CVIM.

**FIGURE 8:**
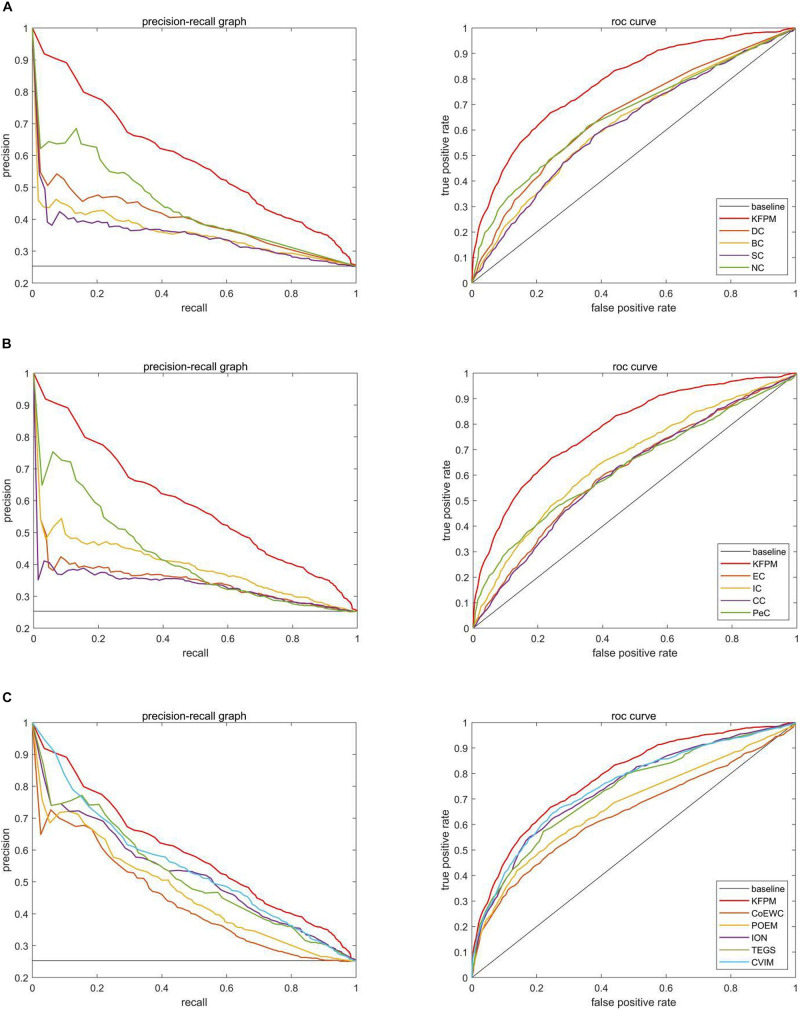
Comparison of PR curves and ROC curves between KFPM and 13 competing methods based on the Krogan database. **(A)** PR curves and the ROC curves of DC, BC, SC, and NC. **(B)** PR curves and ROC curves of EC, IC, CC, and Pec. **(C)** PR curves and ROC curves of CoEWC, POEM, ION, TEGS, and CVIM.

In this section, we will further utilize the ROC (Receiver Operating Characteristic) curve to evaluate the detection performance of KFPM. The closer the ROC curve is to the upper left corner, the higher the recall rate of the model (Hanley and Mcneil, 1982). Figures 7, 8 show ROC curves and PR (Precision Recall) curves of KFPM and 13 competing methods under the DIP and Krogan databases, respectively. As shown in Figure 7, it is obvious that KFPM can achieve better predictive performance than all these 13 state-of-the-art methods based on the DIP database, although the ROC curves of KFPM and CVIM overlap partially in Figure 7C. As shown in Figure 8, it is easy to see that the predictive performance of KFPM is better than all these 13 state-of-the-art methods based on the Krogan database as well. Table 4 shows the superiority of KFPM more intuitively based on the performance indicator of AUCs (Area Under roc Curves).

**TABLE 4:** AUCs achieved by KFPM and 13 competitive methods based on the DIP and Krogan databases.

Method	AUCs (based on DIP)	AUCs (based on Krogan)
DC	0.6704	0.6583
IC	0.6657	0.6573
EC	0.6384	0.6167
BC	0.625	0.6248
SC	0.6384	0.6167
CC	0.6291	0.6114
NC	0.6879	0.6584
Pec	0.6329	0.6316
CoEWC	0.6513	0.6404
POEM	0.6662	0.6726
TEGS	0.7386	0.7287
ION	0.7522	0.7413
CVIM	0.7559	0.7458
KFPM	0.7802	0.7833

Additionally, in order to verify the applicability of KFPM, we further compared KFPM with 13 competitive methods based on the Gavin database. As shown in Table 5, it is easy to see that the prediction performance of KFPM is better than all competing methods, especially, in the top 1% candidate proteins, the number of true essential proteins recognized by KFPM is 19, which means that the recognition rate of KFPM can reach 100%. Hence, we can draw a conclusion as well that KFPM has satisfactory applicability.

**TABLE 5:** The Number of essential proteins recognized by KFPM and 13 competing methods based on the Gavin database.

Methods	Top1%(19)	Top5%(93)	Top10%(196)	Top15%(279)	Top20%(371)	Top25%(464)
DC	7	36	101	158	222	264
IC	16	55	119	163	213	254
CC	11	45	93	135	180	221
BC	9	40	85	122	162	201
SC	0	17	87	130	190	240
EC	0	38	94	134	166	209
NC	11	51	123	170	213	259
CoEWC	16	69	136	190	237	275
Pec	15	69	142	193	238	285
ION	17	73	150	207	263	312
POEM	17	74	148	199	249	296
CVIM	16	80	160	219	271	322
KFPM	19	86	169	216	279	332

### Analysis of Parameters

In KFPM, we have introduced a parameter α ∈ (0,1) to adjust the iterative ratio. Therefore, we will estimate the effect of α on the prediction accuracy of KFPM in this section. Experimental results based on the DIP and Krogan databases are shown in Tables 6, 7 separately. From observing these two tables, it is easy to see that, as a whole, KFEM can achieve the best predictive performance when the value of α is set to 0.5. Moreover, the KFPM can obtain the best performance when the value of θ in formula (15) is set to 0.7.

**TABLE 6:** Influence of the parameter α on the prediction accuracy of KFPM based on the DIP database.

α	0.1	0.2	0.3	0.4	0.5	0.6	0.7	0.8	0.9
**Rank**									
Top1% (51)	47	47	47	48	49	48	47	46	45
Top5% (255)	209	210	211	214	212	210	210	210	210
Top10% (510)	358	363	360	363	360	361	360	363	358
Top15% (764)	466	473	474	477	472	470	465	466	461
Top20% (1019)	572	574	575	570	574	570	566	564	568
Top25% (1274)	647	648	648	648	652	654	653	648	643

**TABLE 7:** Influence of the parameter α on the prediction accuracy of KFPM based on the Krogan database.

α	0.1	0.2	0.3	0.4	0.5	0.6	0.7	0.8	0.9
**Rank**									
Top1% (37)	36	36	35	35	34	34	34	33	31
Top5% (184)	147	147	149	148	147	149	150	149	147
Top10% (367)	264	264	260	257	257	255	254	252	255
Top15% (551)	368	365	361	357	353	348	345	346	339
Top20% (734)	441	441	443	440	435	433	426	422	42
Top25% (918)	499	503	503	504	510	504	505	502	482

## Discussion

Essential proteins are indispensable proteins for the survival and reproduction of organisms. In recent years, identification of essential proteins has become a research hotspot. It takes a lot of time and money to predict the essential proteins through traditional biological experiments. Therefore, many researchers focus on designing effective predictive models by combining PPI networks. With gradual improvement of high-throughput techniques, prediction methods with more accurate predictive performance have been proposed successively based on combination of biological data of proteins and PPI networks. Inspired by this, a novel predictive model called KFPM has been proposed in this paper, which can achieve satisfactory predictive accuracy by combining topological characteristics of a newly constructed protein-domain interaction network and functional characteristics of proteins. Experimental results demonstrate the superiority of KFPM, which may provide a useful tool for future researches on prediction of key proteins.

## Data Availability Statement

The datasets presented in this study can be found in online repositories. The names of the repository/repositories and accession number(s) can be found in the article/Supplementary Material.

## Author Contributions

XH and LW conceived and designed the study. XH, ZC, and LK obtained and processed datasets. XH and LK wrote this manuscript. YT, LW, and LK provided suggestions and supervised the research. All authors contributed to the article and approved the submitted version.

## Conflict of Interest

The authors declare that the research was conducted in the absence of any commercial or financial relationships that could be construed as a potential conflict of interest.
